# Supplementary material for: Development of explicit definitions of potentially inappropriate prescriptions for antidiabetic drugs in people with type 2 diabetes: A Delphi survey and consensus meeting
Source: PLoS One. 2025 Oct 22;20(10):e0334836. doi: 10.1371/journal.pone.0334836 (PMC12543176; doi:10.1371/journal.pone.0334836)
Supplement: Attachment — Submitted filename: Response_to_reviewers.pdf [file pone.0334836.s003.pdf]

Response to Journal Requirement

Dear Editor,

We thank you for your careful review of our manuscript and for highlighting the additional requirements needed to comply with *PLOS ONE* standards. Please find below our detailed responses and the corresponding modifications made in the revised version.

1. PLOS ONE style requirements

We carefully reformatted the manuscript according to the PLOS ONE templates, including file naming conventions, structure, and referencing style.

Modifications:

Section headings (Level 1): increased from 16 pt bold → 18 pt bold

Section headings (Level 2): increased from 12 pt bold → 16 pt bold

Figure formatting: figure titles set in bold

2. Informed consent

We confirm that all participants provided electronic written informed consent before accessing the Delphi survey. They were informed that their responses would remain anonymous and had to actively confirm their agreement by checking a consent box. This procedure was reviewed and validated by the Data Protection Officer of University of Lille. We have clarified this in the Ethics approval of the Methods section.

Modifications:

Lines 109-112 in revised manuscript with track changes, sentence revised from:

"*Participants were informed that their answers would remain anonymous and had to confirm that they did not object to the collection of their personal data*"

To:

"*All participants provided electronic written informed consent before participating in the Delphi survey. They were informed that their answers would remain anonymous and had to actively confirm their agreement by checking a consent box prior to accessing the questionnaire.*"

3. Funding information in manuscript

We removed all funding information from the manuscript body, as requested.

4. Role of funders

We revised the Funding Statement to specify the role of funders. In our case, funders had no role in study design, data collection and analysis, decision to publish, or preparation of the manuscript. The amended Role of Funder statement is also included in the cover letter.

Modifications:

We added the following sentence in the cover letter:

"The funders had no role in study design, data collection and analysis, decision to publish, or preparation of the manuscript."

5. Copyrighted Figure 1 (maps/satellite images)

We confirm that Figure 1 was created *de novo* in R (packages sf and tmap) using only openly licensed, with no Google or other proprietary basemaps. Country boundaries were obtained from Natural Earth (public domain). We have added a sentence in the Methods to document these sources and licenses. This complies with PLOS ONE's CC BY 4.0 requirements.

Modifications:

Lines 183-184 in revised manuscript with track changes, we added the sentence follow:

"Fig. 1 was generated in R (sf, tmap) using Natural Earth administrative boundaries (public domain). No proprietary basemaps were used."

6. Captions for Supporting Information

We added full captions for all Supporting Information files at the end of the manuscript and ensured that in-text citations match.

7. Reviewer-suggested citations

We carefully evaluated the suggested references. Relevant works have been cited in the revised manuscript; non-essential references were not added, in line with PLOS ONE's policy.

8. Reference list

We thoroughly reviewed the reference list for completeness and accuracy. No retracted articles were cited.

Additional editor request: Grammar

We thank the editor for highlighting the importance of ensuring correct grammar. To address this, we have had the entire manuscript reviewed and edited by a professional scientific editor (David Fraser, Biotech Communication).

Response to Reviewer 1 Comments

We thank Reviewer 1 for comments and constructive suggestions. We have carefully revised the manuscript according to each comment and suggestion. In the following, our responses are given in blue.

Comment: *The paper was generally well written. Background: Captures the essentials of what the survey is about and the general aim of the paper. Methodology: Sound. Results and Discussion: Presented in a logical and simplified manner. Conclusion: It is a reflection of the aims and objectives that the authors sought to obtain. Authors should declare if they received funding or not for this work.*

We sincerely thank the reviewer for the positive evaluation of our manuscript.

Regarding the funding declaration, we confirm that this research was supported by PreciDIAB, which is jointly funded by the French National Agency for Research (ANR-18-IBHU-0001), the European Union (FEDER – agreement NP0025517), the Hauts-de-France Regional Council (agreement 20001891/NP0025517), and the European Metropolis of Lille (MEL, agreement 2019_ESR_11). The funding statement has also been amended according to the Editors' comments and PLOS ONE policy.

Modifications:

In accordance with PLOS ONE requirements, we have removed this information from the manuscript and ensured that it appears only in the Funding Statement section of the submission system. We have also included the following mandatory sentence on the role of funders in our cover letter:

"The funders had no role in study design, data collection and analysis, decision to publish, or preparation of the manuscript."

Response to Reviewer 2 Comments

We thank Reviewer 2 for comments and constructive suggestions. We have carefully revised the manuscript according to each comment and suggestion. In the following, our responses are given in blue.

INTRODUCTION

Comment: *Acknowledge that neither an implicit nor explicit approach to reducing potentially inappropriate medicines is a "fix all".*

We agree with the reviewer's observation. It is obvious for all co-authors, but we agree that it should be written in an explicit way in the introduction. To address this, we have added a sentence in the Introduction to explicitly acknowledge that neither implicit nor explicit approaches can serve as a universal solution to inappropriate prescribing.

**Modifications:**<br><br>Lines 65-66 in revised manuscript with track changes, we added the following sentence in the revised manuscript with track changes:<br><br>"*However, neither implicit nor explicit approaches should be considered as a universal solution to inappropriate prescribing.*"

Comment: *Include reference to personalization of treatment, which was inferred but not explicit.*

We agree that personalization of treatment is an important concept and have revised the Introduction to explicitly highlight the importance of individualizing therapy according to patient characteristics.

**Modifications:**<br><br>Lines 66-67 in revised manuscript with track changes, we added:<br><br>"*Implicit and explicit approaches should be combined, which underlines the importance of treatment personalization according to individual patient characteristics* [1, 13]."

Comment (Line 57): "*physicians and pharmacists face barriers to implementing guidelines…*"*- be explicit that you are referring to national and international guidance on prescribing of ADs.*

We thank the reviewer for this suggestion. We have clarified the text to explicitly state that the barriers mentioned relate to the implementation of both national and international guidelines.

**Modifications:**<br><br>Line 56 in revised manuscript with track changes, sentence revised from:<br><br>*"Physicians and pharmacists face barriers to implementing guidelines on the prescribing of antidiabetic drugs."*<br><br>to:

"Physicians and pharmacists face barriers to implementing ***national and international*** *guidelines on the prescribing of antidiabetic drugs."*

METHODS

Comment (Lines 86–87): *Please include that the explicit definitions were identified by literature as described in the study protocol.*

We have clarified this point by specifying that explicit definitions were identified through a literature review and a qualitative study as outlined in the study protocol.

Modification:

Lines 96-98 in revised manuscript with track changes:

"The explicit definitions were identified through a systematic review of the literature and qualitative study, as described in the study protocol."

Comment (Lines 106–107): *How were these participants identified in the first instance? Were they from existing relationships within the steering group or did you reach out to special interest groups, etc.*

Participants were identified through the professional networks of the steering committee. Each steering committee member reached healthcare professional who were specifically invested in AD prescribing and then contacted them by email.

Modifications:

Lines 118-121 in revised manuscript with track changes, sentence revised from:

"Participants were recruited via email."

To:

"Participants were identified through the professional networks of the steering committee. Each steering committee member reached healthcare professional who were specifically invested in AD prescribing and then contacted them by email."

Comment (Line 117): *"the participants had to complete a questionnaire and indicate" refine to "participants completed a questionnaire and indicated.."*

We have revisited the sentence as suggested.

Modifications:

Line 132 in revised manuscript with track changes, sentence revised from:

"The participants had to complete a questionnaire and indicate..."

To:

“Participants completed a questionnaire and indicated...”

Comment (Lines 122–132): *Were participants provided with an individual report and the start of round 2 to inform them how their responses compared to others participating in the survey? Did you apply analyses e.g. a mixed effects regression model to the data. If not, why not?*

We appreciate this question and have clarified our procedure. Participants did not receive individual feedback reports between rounds. Instead, for all items advanced to round 2, we presented aggregate feedback together with a synthesis of arguments during the first consensus meeting. This format enabled deeper discussion and more reflective re-assessment; colleagues who could not attend consensus meeting received the same quantitative summaries and a written synopsis of the discussion. To limit respondent burden, we deliberately did not embed these materials in the round-2 online questionnaire. We deliberately favored qualitative exchange and structured discussion over regression-based quantitative analyses, which, in our experience from previous Delphi studies, have limited impact on participants' decisions. (https://pubmed.ncbi.nlm.nih.gov/38534718/, https://pubmed.ncbi.nlm.nih.gov/29433501/)

Modification:

We add the following sentence in lines 135-139 in revised manuscript with track changes *“Participants did not receive individual feedback reports between rounds. For items carried forward to round 2, we presented aggregate feedback together with a brief synthesis of arguments during the first consensus meeting to support reflective re-assessment. To minimise respondent burden, these materials were not embedded in the round-2 online questionnaire; non-attendees received the same quantitative summaries and a written synopsis of the discussion.”*

Comment (Lines 155–156): *"In other cases, the explicit definitions were considered to be indeterminate (neither validated156 nor rejected have been neither e in an state"... this sentence does not make sense.*

We thank the reviewer for pointing out this ambiguity. The sentence has been rewritten to clearly explain that certain explicit definitions were considered indeterminate when neither validated nor rejected.

Modification:

Line 177 in revised manuscript with track changes, sentence revised from:

“In other cases, the explicit definitions were considered to be indeterminate (neither validated nor rejected have been neither in an state)”

To:

*“In other cases, the explicit definitions were considered indeterminate **(meaning that they were neither validated nor rejected by the panel**).”*

RESULTS

Comment (Table 1): *Median year of qualification incorrectly reported. The table reports the median year, not the median years since qualification.*

We agree with the reviewer and have corrected this error to accurately reflect that the table reports the median year of qualification, not the number of years since qualification.

Modifications:

In Table 1, we revised the label from:

"Median years since qualification [range]"

To:

*"Median year **of** qualification [range]"*

Comment (Figure 1): *Figure 1. Whilst this is interesting to see which areas of France were represented in the Delphi study, it would also be interesting to see which healthcare professionals from these regions also participated. you report that you have one Swiss and one Belgian participant, but it would have been interesting to see what type of healthcare professional this was, e.g. GP, community pharmacist, etc.*

We thank the reviewer for this suggestion. We have revised Figure 1 to include the distribution of participants by professional background (general practitioners, diabetologists, community pharmacists, hospital pharmacists, pharmacologist) and specified the professional category of the Swiss and Belgian participants.

Modification (Figure 1):

Figure 1. Geographic distribution of Delphi participants

Abbreviations: GP = general practitioner; Diab = diabetologist; C-Pharm = community pharmacist; H-Pharm = hospital pharmacist; Pharmacol = pharmacologist.

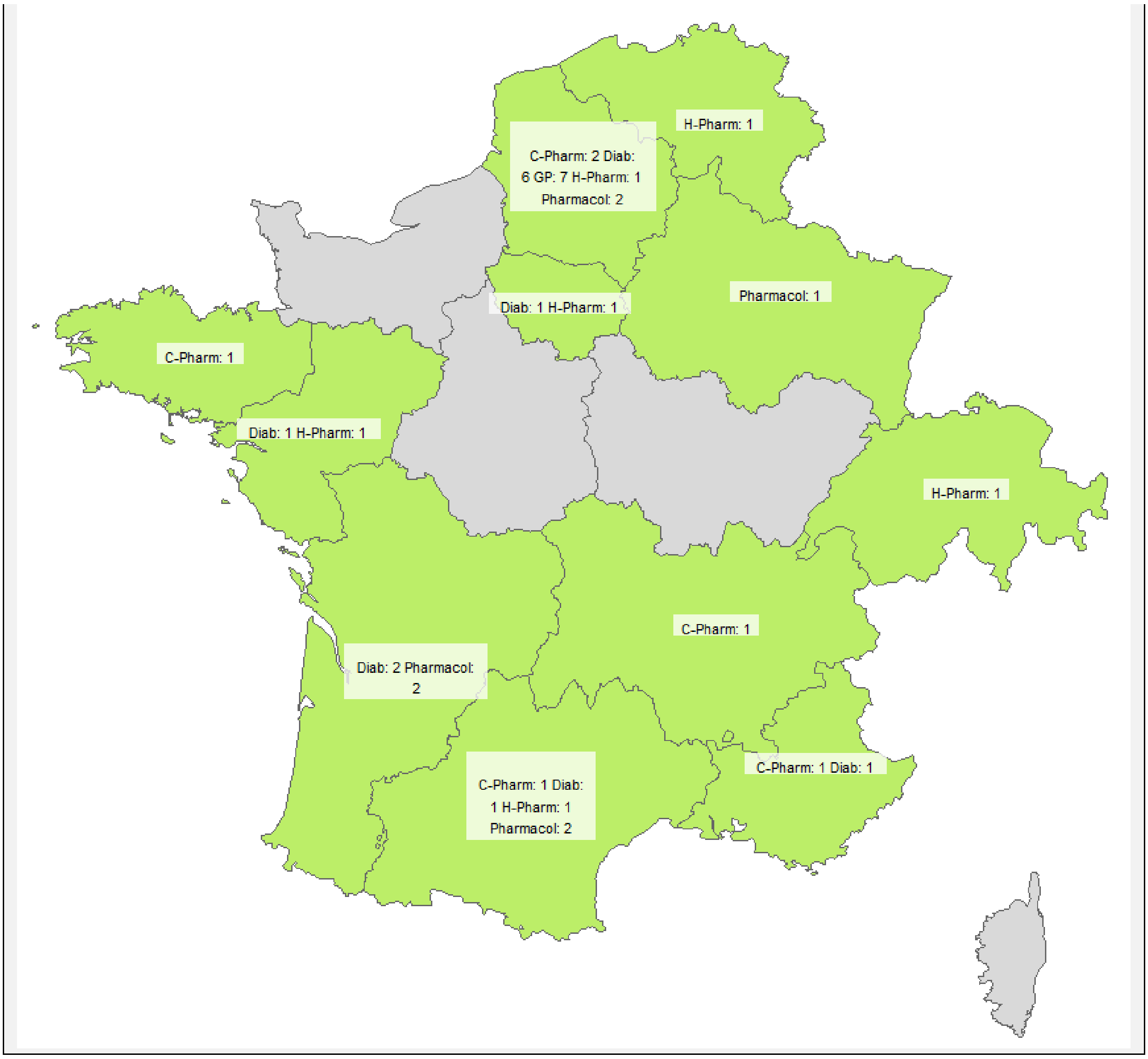

Comment (Line 175): *I would open this paragraph describing figure 2 and then outline the timeframe. Currently the reference style made me think the figure also contained dates and not the flow of participants and the evolution of the definitions.*

We have restructured the paragraph so that it first introduces Figure 2 before describing the timeframe of the Delphi process.

Modifications:

Lines 200 in revised manuscript with track changes, we added:

"Fig. 2 illustrates the flow of participants across the two Delphi rounds and the evolution of the explicit definitions over time."

Comment (Line 183): *Space required between the end of one sentence and start of another.*

We corrected the typographical error by inserting the missing space.

Modifications:

Line 209 in revised manuscript with track changes revised from:

"2024.Of..."

To:

"2024. Of..."

DISCUSSION

Comment (Line 217): "*Polypharmacy is very prevalent"- do not need "very"*.

We agree and have simplified the sentence by removing the unnecessary qualifier.

Modifications:

Line 275 in revised manuscript with track changes revised from:

"Polypharmacy is very prevalent among older adults with diabetes."

To:

"Polypharmacy is prevalent among older adults with diabetes."

Comment (Line 225): "E*xplicit tools for use with populations of older adults have been studied extensively"- give examples*

We have added examples of widely used explicit tools, including the Beers criteria, STOPP/START, and the EU(7)-PIM list with references.

Modifications:

We modified Line 265 in revised manuscript with track changes as suggested:

"For example, the Beers criteria, STOPP/START, and the EU(7)-PIM list are widely used explicit tools for older adults [24, 25, 44-47]."

Comment (Lines 232–250): *It feels more organic to lead with existing PIP tools then your tool, highlighting the differences.*

We thank the reviewer for this constructive suggestion. We have reorganized the paragraph in the Discussion so that it now begins by describing existing explicit PIP tools (Beers, STOPP/START, EU(7)-PIM, PRISCUS), their benefits, and evidence of their impact. We then introduce our Delphi-based explicit list for antidiabetic drugs, situating our work as a complementary contribution in this established field. This reorganization improves the logical flow and makes the novelty of our contribution clearer.

Modifications:

Lines 264-283 in revised manuscript with track changes revised from:

"Our research study was prompted by studies in the field of geriatrics, in which PIPs were detected with explicit tools [24, 25, 40–43]. The explicit list obtained is specific for the management of ADs (excluding insulin) for people living with T2DM. Polypharmacy is very prevalent in people living with T2DM, due to the many comorbidities. Hence, the management of AD prescriptions in primary care can be challenging [5]. Faquetti et al.'s study of a primary care cohort in the UK showed that 39.6% of people with T2DM aged 65 or over had one or more PIPs of non-insulin ADs [44]. It has been shown that PIP-ADs are associated with a greater risk of adverse drug reactions, such as hypoglycaemia, pancreatitis, and ketoacidosis [45–47]. This new list of PIP-ADs might notably be of help to healthcare professionals who manage people living with T2DM, and especially professionals who are not diabetes specialists or when patients are managed in an isolated, non-multidisciplinary context. Explicit tools for use with populations of older adults have been studied extensively. For example, the Beers criteria, STOPP/START, and the EU(7)-PIM list are widely used explicit tools for older adults [48, 49]. The use of lists of explicit PIPs is known to improve the appropriateness of prescriptions [26], decrease the risk of adverse drug reactions [50], reduce healthcare costs [51], and increase health-related quality of life [52]. These benefits have been observed in primary care [53–55], in nursing homes [56, 57] and in hospital settings [26, 58, 59]. These studies also highlighted the importance of collaboration between the various healthcare professionals involved in patient care, i.e. general practitioners, pharmacists, and nurses."

To:

"Explicit tools for use with populations of older adults have been studied extensively, such as the Beers criteria, the STOPP/START criteria, the EU(7)-PIM list, and the PRISCUS list [24, 25, 40, 43, 48, 49]. The use of such lists is known to improve the appropriateness of prescriptions [26], decrease the risk of adverse drug reactions [50], reduce healthcare costs [51], and increase health-related quality of life [52]. These benefits have been observed in primary care [53–55], in nursing homes [56, 57] and in hospital settings [26, 58, 59]. These studies also highlighted the importance of collaboration between the various healthcare professionals involved in patient care, i.e., general practitioners, pharmacists, and nurses.

Building on this background, our research study was prompted by the need to develop explicit tools specific to the management of ADs. The explicit list obtained in the present study is specific for the management of ADs (excluding insulin) for people living with T2DM. Polypharmacy is prevalent in this population, due to the many comorbidities, and the management of AD prescriptions in primary care can be challenging [5]. Faquetti et al.'s study of a primary care cohort in the UK showed that 39.6% of people with T2DM aged 65 or over had one or more PIPs of non-insulin ADs [44]. It has been shown that PIP-ADs are associated with a greater risk of adverse drug reactions, such as hypoglycaemia, pancreatitis, and ketoacidosis [45–47]. This new list of PIP-ADs might notably be of help to healthcare professionals who manage people living with T2DM, especially professionals who are not diabetes specialists or when patients are managed in an isolated, non-multidisciplinary context."

Comment (Lines 259–262): *how can this limitation be adapted for an international audience?*

We have clarified that, similar to other tools, our definitions should be adapted to international contexts by considering differences in drug availability and prescribing practices.

Modifications:

Lines 328-329 in revised manuscript with track changes, we added:

"Our definitions should be adapted to international contexts by considering differences in drug availability and prescribing practices."

Comment (Line 273): *These seems rather extensive. Other Delhi publications have thanked the participants collectively.*

We respectfully acknowledge the reviewer's suggestion. However, we prefer to maintain a more detailed Acknowledgments section. In a Delphi process, the contribution of participants is central to the validity of the consensus, and we believe it is important to recognize their efforts more explicitly. Listing the participants individually not only ensures transparency but also appropriately acknowledges their investment of time and expertise in multiple survey rounds. We therefore propose to keep the current format, while making minor adjustments for conciseness if required by the editor.

Response to Reviewer 3 Comments

We thank Reviewer 3 for comments and constructive suggestions. We have carefully revised the manuscript according to each comment and suggestion. In the following, our responses are given in blue.

INTRODUCTION

Comment 1: *Briefly mention the clinical consequences of PIP-ADs (e.g., hypoglycemia, ketoacidosis) earlier to strengthen rationale.*

We thank the reviewer for this helpful suggestion. We have revised the Introduction to mention the clinical consequences of potentially inappropriate prescribing of antidiabetic drugs (PIP-ADs) earlier, to strengthen the rationale for our study.

Modifications:

Lines 73-74 in revised manuscript with track changes, we added the sentence as follows:

"PIP-AD can lead to serious clinical consequences such as hypoglycemia, ketoacidosis, or pancreatitis, underlining the importance of developing AD-specific criteria."

Comment 2: *Clarify why insulin was excluded from the scope (is it due to different prescribing dynamics?).*

Thank you for raising this point. Insulin was a priori excluded (decision made in 2020) because its prescribing and dose titration are highly individualized and often require day-to-day adaptations based on patient-specific information (e.g., recent HbA1c, capillary/CGM glucose profiles, hypoglycaemia history, intercurrent illness, comorbidities, and patient preferences). Such dynamics are difficult to translate into standardized explicit criteria suitable for a Delphi rule set, whereas non-insulin antidiabetic drugs can be operationalized more consistently. This rationale is consistent with contemporary guidance emphasizing individualized insulin regimens (e.g., ADA/EASD consensus; practical insulin titration guidance). We have therefore added a sentence to clarify that insulin prescriptions follow different dynamics, with individualized regimens that are difficult to capture in explicit criteria.

Modifications:

Lines 82-84 in revised manuscript with track changes, we added the sentence as follows:

"In this study, we focused on non-insulin antidiabetic drugs. Insulin was excluded because its prescribing is highly individualized, requiring titration and frequent patient-specific adjustments, which makes it less suitable for standardized explicit definitions."

METHODS

Comment 3: *The target distribution (25% GPs, 25% diabetologists, etc.) is appropriate, but the final sample slightly deviates (e.g., 18.4% GPs). A brief discussion on whether this affected results would be helpful.*

We thank the reviewer for pointing this out. We have added a note in the Results section to acknowledge that although the final distribution of participants differed slightly from the initial target, the diversity of professional backgrounds was preserved. We consider that this did not substantially affect the validity of the results.

Modifications:

Lines 317-319 in revised manuscript with track changes, we added the sentence as follows:

"Although the final distribution of participants differed slightly from the initial target (e.g., 18.4% GPs instead of 25%), the panel remained multidisciplinary, ensuring that the consensus reflected the perspectives of all major professional groups."

Comment 4: *Geographic distribution (mostly France) may limit generalizability. Acknowledging this as a limitation is sufficient.*

We agree with the reviewer. We have added a sentence in the Discussion (limitations section) acknowledging that the predominance of French participants may limit generalizability, and that future studies should validate these explicit definitions in other countries.

Modifications:

Lines 328-329 in revised manuscript with track changes, we added the sentence as follows:

"Our definitions can be adapted to international contexts by considering differences in drug availability and prescribing practices."

Comment 5: *The 75% agreement threshold is standard, but was there any consideration of weighting responses by expertise (e.g., diabetologists vs. pharmacists)?*

We confirm that all participants' responses were weighted equally, regardless of expertise, as this approach is consistent with the Delphi methodology. We have clarified this point in the Methods section.

Modifications:

Lines 188-189 in revised manuscript with track changes, we added the sentence as follows:

"All participants' responses were weighted equally, irrespective of professional expertise, in line with standard Delphi methodology."

<u>RESULTS</u>

Comment 6: *Table 1 is clear, but the female percentage for GPs (14%) seems unusually low. Is this representative of the GP population in the region?*

We acknowledge that the proportion of female GPs (14%) in our sample is lower than in the general GP population. This discrepancy reflects the composition of the recruited sample and may not be fully representative of the GP population in France. However, we do not believe this substantially affects our results, since the Delphi process is designed to capture expert

consensus across professional groups rather than to provide a statistically representative sample of the population.

Modifications:

Lines 319-323 in revised manuscript with track changes, we added the sentence as follows:

"The proportion of female GPs (14%) reflects our recruited sample and may not be fully representative of the GP population in France. Nevertheless, this discrepancy is unlikely to have influenced the validity of our findings, as the Delphi methodology aims to synthesize expert opinions rather than to achieve population-level representativeness."

Comment 7:

Some definitions (e.g., #35 on metformin and dehydration) are highly specific. Were these derived from real-world cases or guidelines? A brief rationale for inclusion would help.

We confirm that highly specific definitions, such as the case of metformin and dehydration, were not arbitrarily added but originated from the combination of two sources: (1) proposals collected during the preliminary qualitative study, and (2) suggestions refined and validated during the Delphi rounds. These proposals were often aligned with, or inspired by, recommendations already present in national and international guidelines. We have clarified this point in the Results section.

Modifications:

Lines 238-242 in revised manuscript with track changes, we added the sentence as follows:

"Some definitions were highly specific (e.g., the combination of metformin and dehydration). These originated from the proposals collected during the preliminary qualitative study [32]) and from expert contributions during the Delphi rounds, and were subsequently discussed during the consensus meetings to ensure alignment with national and international guidelines."

DISCUSSION

Comment 8:

Clinical Relevance: Discuss how frequently these PIP scenarios occur in practice (e.g., are some rare?).

We agree that estimating how often each specific PIP-AD definition occurs in practice is important. To our knowledge, there are no published prevalence estimates for most individual combinations covered by our list. We therefore added a cautious statement in the Discussion: some scenarios are likely uncommon, whereas others (e.g., sulfonylurea exposure in older adults) appear frequent in routine care. We note that in our own ongoing work in older adults we observed a non-negligible rate for one repaglinide-related definition; however, as these data are under review, we did not cite them in the manuscript.

Modifications:

Lines 242-243 in revised manuscript with track changes, we added the sentence as follows:

“While certain PIP scenarios may be uncommon, others (e.g., sulfonylurea use in older adults) are frequent in routine practice, reinforcing the importance of clear explicit definitions.”

Comment 9:

How feasible are these definitions for automated CDSS use? Some may require complex data (e.g., HbA1c trends, patient-reported symptoms)

We agree that the feasibility of automation depends less on the logic of the explicit definitions than on the availability and granularity of the underlying EHR data. For example, a rule such as “Drug A inappropriate in Condition B” Is, in principle, machine-readable; however, its performance will vary with (i) how drugs are identified (active-ingredient or RxNorm/ATC 5th-level mapping versus broader ATC class matching, which may increase recall at the cost of false positives), and (ii) how conditions are encoded (specific ICD-10/SNOMED codes versus symptoms or free text). Several definitions also require structured laboratory data (e.g., eGFR, HbA1c) and explicit time windows. Our Lille team has extensive experience with CDSS in hospital settings (https://pubmed.ncbi.nlm.nih.gov/34042654/, https://pubmed.ncbi.nlm.nih.gov/39558377/)

We have expanded the Discussion to acknowledge these implementation choices and to recommend publishing local code lists and performing calibration/validation to balance sensitivity and precision.

Modifications:

Lines 290-295 in revised manuscript with track changes, we added the sentence as follows:

“Explicit definitions are machine-readable, but CDSS automation depends on local data and coding. Rules based on structured EHR fields (e.g., age, comorbidities, laboratory results) can be implemented directly, whereas performance varies with how drugs and conditions are encoded (active-ingredient vs ATC ATC 5th-level mapping, specific ICD-10 or SNOMED codes). By contrast, concepts such as dehydration or recurrent hypoglycaemia may exist only in free text, which limits automation.”

Comment 10 (Discussion – Limitations):

Acknowledge that the list may not cover all ADs (e.g., thiazolidinediones, as noted). The study focused on non-insulin ADs—could future work include insulin?

We agree. We now state that our list does not yet cover all non-insulin antidiabetic classes and that insulin was intentionally excluded because its dosing is highly individualized and increasingly mediated by integrated patient-level technologies (insulin pumps, hybrid closed-loop/automated delivery, CGM-driven adjustments), which are difficult to encode as fixed explicit rules. Future work will extend coverage to additional classes and explore narrowly defined, objective insulin-related criteria.

Modifications:

Lines 331-333 in revised manuscript with track changes:

"Another limitation is that the list does not cover all antidiabetic drug classes (e.g., thiazolidinediones) and excludes insulin. Future research should extend these explicit definitions to a broader range of drugs, including insulin."

CONCLUSION

Comment 11:

Consider adding a sentence on next steps (validation in real-world settings).

We agree with the reviewer. We have added a sentence in the Conclusion emphasizing the importance of real-world validation.

Modifications:

Lines 341-343 in revised manuscript with track changes: we added the sentence as follows:

"The next step will be to validate these explicit definitions in real-world clinical settings to assess their feasibility, reliability, and impact on prescribing practices, particularly in the context of clinical decision support systems."
